# Determining the factors affecting the gestational length in sheep

**DOI:** 10.5194/aab-64-83-2021

**Published:** 2021-02-23

**Authors:** Hilal Tozlu Celik, Fatih Ahmet Aslan, Yeliz Kasko Arıcı, Metehan Eser Kahveci, İbrahim Kiper

**Affiliations:** 1 Department of Food Processing, Ulubey Vocational School, Ordu University, 52850 Ulubey, Ordu, Turkey; 2 Department of Chemical and Chemical Processing Technologies, Ulubey Vocational School, Ordu University, 52850 Ulubey, Ordu, Turkey; 3 Department of Basic Medical Sciences, Faculty of Medicine, Ordu University, 52000 Altınordu, Ordu, Turkey; 4 Veterinary Department, Ulubey Vocational School, Ordu University, 52850 Ulubey, Ordu, Turkey; 5 Ordu Sheep and Goat Breeders' Association, 52000 Altınordu, Ordu, Turkey

## Abstract

This research aimed to determine the effects of body weight, the body condition score (BCS), body measurements, birth type (single offspring or twin birth), birth weight and sex on the gestational length in sheep (n=111). Karayaka sheep raised on a private farm were used in the study. Progeny yield characteristics in sheep were also determined (n=139). Estrus was monitored daily using teaser rams from August to September 2016. According to our findings, the pregnancy rate, infertility rate, fecundity, and twin and single birth rates were 93.52 %, 6.48 %, 93.52 %, 14.62 % and 85.38 % respectively. The effect of age on the BCS in sheep at mating was found to be significant (P<0.05): BCS decreased as age increased. It was determined that there was a positive association between the BCS and live weight during the mating period (P<0.001). The chest circumference, front shin circumference and body length were found to be higher in sheep with a BCS ≥4.5 at mating time (P<0.01). The middle rump width was significantly affected by the BCS (P<0.001). In this study, the lowest and highest gestational lengths were found to be 148.90 and 151.41 d respectively. The gestational length in sheep was not found to be affected by age, the BCS, body measurements, sex or birth type (P>0.05); however, it was observed that the gestational lengths for male offspring and single offspring (non-multiple births) were longer. In addition, it was detected that the gestational length was different in sheep with a BCS ≥4.5. The time spent in the womb is important with respect to obtaining a healthy lamb. For profitable production, a BCS of between 2.5 and 4 is recommended in sheep. It is thought that the use of body condition scoring, which is easy information for the breeder to utilize, will have a positive effect on determining the bodily reserves of sheep and the reproductive efficiency as well as on obtaining a healthy lamb. More studies on the gestational length in sheep are required.

## 1 Introduction

Karayaka sheep are usually bred in the Black Sea region (Ordu, Giresun, Samsun, Sinop and Tokat provinces) and constitute 4.61 % of the total sheep population in Turkey (Turkish Statistical Institute, 2020). Karayaka sheep are a breed with a low milk and offspring yield, a fine tail and a coarse mixed fleece. They are known to have good-quality meat. In Karayaka sheep, the number of lambs at birth is 1.05 (Akcapınar et al., 2002). For this reason, it is beneficial for breeders to increase the fertility rate in this breed via various breeding methods (Cam et al., 2017). The period spend in the uterus has an important effect on the development of the offspring. In Karayaka sheep, the pregnancy rate, return rate, twinning rate and triplet rate of females from twin births mated with rams from twin births were determined to be 100, 16.22, 52.25 and 3.60 respectively. Thus, the selective breeding of twin animals in Karayaka sheep may increase the rate of twinning (Cam et al., 2017).

The body condition score (BCS) can vary depending on the environment and husbandry conditions (Özdemir, 2008). The gestational length in sheep is influenced by the interaction of maternal age, offspring sex, genotype, nutrition, environmental temperature, year, lambing season, location of the enterprise and geographical location. It is stated that approximately two-thirds of the variation seen among sheep breeds in terms of gestational length is caused by the genotype of the fetus (Ates et al., 2003; Ahmad and Khan, 2008; Cedden, 2002; Koyuncu et al., 2001; Koyuncu and Duru, 2003; Odabaşıoğlu et al., 1996). The average gestational length of Kari sheep in Pakistan has been determined to be 110.2±1.10 d. It is stated that this situation is caused by the genetic structure (Ahmad and Khan, 2008). The mean gestational length was found to be 150.6±0.64 d in Nigerian sheep and their crosses, and it was not affected by sex or birth type (single offspring or twin birth; P>0.05). During pregnancy, nutrition, BCS, body measurements and body weight affect the offspring, as does the number of ovarian cells in the mid-gestational period, although these effects may decrease at the end of pregnancy (Asmad et al., 2015). It has been reported that the gestational length was shorter in fertile breeds (Öztürk and Aktaş, 1996). A longer gestational length was also found to increase the lamb birth weight (Ates et al., 2003). It was determined that the gestational length in Kari sheep in Pakistan can vary between 3 and 5 months (Ahmad and Khan, 2008). It has been stated that the pregnancy rate is low in overweight sheep, whereas the abortion rate is high (Staykova et al., 2013). The gestational length in Nigerian sheep was determined to be 151.6 d for male offspring, 150.0 d for female offspring, 150.5 d for single offspring births, 151.0 d for twin births and 150.6 d for the herd average (Iyiola-Tunji et al., 2010). It was determined that lambs with high birth weights spent more time in the uterus (Öztürk and Aktaş, 1996).

The BCS is a method that can be easily applied by the breeder and gives information on the nutritional status of the animals. At the same time, it is a system based on the grading of the differences that can be observed in the fattening of the organism with the help of identifiable physical properties. Sheep in good condition during the mating period show a higher value in terms of reproductive efficiency. Determining the BCS of the herd and bringing it to an optimum level during the mating period in sheep increases the number of lambs. The body weight and BCS at the time of mating have positive effects on some reproductive traits, such as ovulation rate and the number of lambs born (Atti et al., 2001; Vatankhah et al., 2012; Kandemir et al., 2013; Van Der Linden et al., 2014). At the same time, the mother's bodily reserves have an effect on the lamb's feeding and on the mother's mammary glands after birth (Van Der Linden et al., 2014). In Karya-type sheep, the average respective BCS and body weight values during the mating period were found to be 1.85±0.06 and 42.95±0.66kg (Özdemir, 2008). The BCS in Caucasian Merino sheep was affected by the physiological condition. Pregnancy rates were found to be higher in animals with a BCS between 2.5 and 3.5, and the abortion rate was higher in those with a BCS < 2 (Staykova et al., 2013). Fetal development was negatively affected in sheep that were malnourished at the beginning of pregnancy, animals with a low body weight and animals carrying twin offspring (Asmad et al., 2015). A BCS of between 2.5 and 3.5 during the mating period positively affected the lambing rate (Staykova et al., 2013). Specifically, more efficient use of limited feed resources, via utilization of the BCS, during the breeding season can increase the total income by increasing the reproductive yield and the number of lambs obtained (Yılmaz et al., 2011; Sejian et al., 2015). The BCS differs among diverse breeds and ages in sheep (Türkyılmaz et al., 2017). It is also an important indicator of the milk yield of lactating ewes after birth and, therefore, of adequate nutrition of the offspring. Nutrition during pregnancy is necessary for both the development of the offspring and the needs of the mother. It is known that fertility in Karayaka sheep is low; however, it can be improved by controlling environmental effects and by undertaking selective breeding studies (Cam et al., 2017).

In this study, the effects of age, BCS, body weight and body measurements, birth weight, sex, and birth type on the gestational length were examined. It is thought that the results obtained will contribute to expanding upon existing knowledge in the literature.

## 2 Material and methods

This study was carried out in accordance with the ethical principles and rules of decision number 1 of the Ordu University Animal Experiments Local Ethics Committee dated 27 January 2016.

### 2.1 Data set

The research was carried out on Karayaka sheep raised on a private farm in Bolaman Gölbaşı village in the Fatsa district of Ordu. Animals were selected for the study, their ear tag numbers and ages were recorded, and their reproductive yield records were evaluated (n=139). Data were analysed for the factors (body condition score, age, live weight, body measurements, birth type, sex and birth weight) affecting the gestational length of 111 pregnant ewes, according to the BCS values obtained during the mating period.

### 2.2 Animal management

The sheep were grazed in the hazelnut garden in the village of Bolaman and were taken to Çambaşı Plateau, Sinanlıobas, Ordu, as the weather got warmer. The animals were grazed on the pasture between 06:00 and 13:00 LT and between 14:00 and 20:00 LT. During the pregnancy period, approximately 600 g of a corn, barley and wheat mixture was given to each female per day. After the sheep returned from the plateau, they were again grazed in the hazelnut garden. The breeder did not intervene in the animals' nutrition. Lambs were weaned on the 90th day after birth.

### 2.3 Mating

The mating period took place between 2 August and 9 September 2016. Four weeks before the mating period, the sheep were weighed using an animal scale with a sensitivity of 100 g, and their live weight was determined. Body condition scores and body measurements were also taken. Their body condition was scored using the 0.5 scale (1 to 5 scale) defined by Russel et al. (1969). The ewes were divided into four groups based on their BCSs: BCS≤2.5 (n=27), BCS between 2.5 and 3.49 (n=42), BCS between  3.5 and 4.49 (n=32) and BCS≥4.5 (n=10). During the mating period, mating dates and the ear tag numbers and ages of the females and rams were recorded. During the same period, certain body measurements were taken. The gestational length was calculated by recording the mating dates of the ewes and the birth dates of the lambs. Abortion was not detected during pregnancy. The lambs were weighed and ear-tagged after birth. The birth type and sex of the lambs were recorded.

### 2.4 Statistical analysis

The data were tested for normality using a Kolmogorov–Smirnov test and for homogeneity of variance using a Levene's test prior to the analyses. Categorical variables were analysed using the Pearson $\chi^{2}$ test. Continuous variables were analysed with an independent sample t test or one-way ANOVA followed by a Tukey post hoc test.

## 3 Results

Data on the reproductive efficiency characteristics of Karayaka sheep from this study are given in Table 1. According to the findings, the pregnancy rate, infertility rate, fecundity, twinning rate, single birth rate and survival rate of lambs during the weaning period were 93.52 %, 6.48 %, 93.52 %, 14.62 %, 85.38 % and 83.85 % respectively. The litter size at birth and weaning were 1.00 and 0.78 respectively (Table 1).

**Table 1:** Descriptive information on the reproductive traits the Karayaka sheep from this study.

Traits	n
Number of sheep during the mating period	139
Pregnant sheep	130
Pregnancy rate	93.52 %
Infertile sheep	9
Infertility rate	6.48 %
Aborted pregnancies	0
Lambs born	130
Fecundity	93.52 %
Number of twin births	19
Twinning rate	14.62 %
Number of single births	111
Single birth rate	85.38 %
Number of lambs born from each ewe that mated	0.94
Litter size at birth	1.00
Stillbirths	6
Number of lambs weaned	109
Percentage of lambs that survived the weaning period	83.85 %
Litter size at weaning	0.78
Number of weaned lambs per ewe that gave birth	0.84

The results obtained during the mating period are given in Table 2. The effect of age on the BCS in sheep was found to be significant (P<0.05): the BCS decreased as age increased. The increase in sheep age caused a decrease in the BCS and live weight (LW). It was determined that there was a positive association between the BCS and LW during the mating period (P<0.001). It was also determined that the chest circumference (CC), front shin circumference (FSC) and body length (BL) were larger in females with a BCS ≥4.5 during the mating period, and the effect of the BCS on the CC, FSC and BL was found to be significant (P<0.01). The withers height (WH) and rump height (RH) were not affected by the BCS (P>0.05). However, the middle rump width (MRW) was significantly affected by the BCS (P<0.001). The middle rump widths of sheep with a BCS between 3.5 and 4.5 and >4.5 were different from sheep with a BCS<2.5 and between 2.5 and 3.49. As the BCS increased, the middle rump widths increased. The gestational length was not affected by BCS changes in Karayaka sheep (P>0.05). In this research, the lowest gestational length was found to be 148.90 d, whereas the highest was found to be 151.41 d. It was determined that the single birth rate of Karayaka sheep was higher than the twin birth rate. It was also found that more than half of the offspring born were female. The lamb birth weight was not affected by BCS during the mating period (Table 2).

**Table 2:** Descriptive statistics and comparison results for breeding season variables in terms of the four BCS groups. The results are given as the mean ± SD (standard deviation).

Variables	BCS groups				*P*
	<2.5 (n=27)	2.5–3.49 (n=42)	3.5–4.49 (n=32)	≥4.5 (n=10)	
Sheep age	$4.44\pm 2.06^{a}$	$3.67\pm 1.46^{\text{ab}}$	$3.19\pm 1.42^{b}$	$3.80\pm 1.14^{\text{ab}}$	0.031${}^{*}$
Sheep body weight at mating	$54.46\pm 5.10^{c}$	$57.24\pm 4.61^{\text{bc}}$	$58.90\pm 5.18^{b}$	$65.07\pm 4.88^{a}$	0.000${}^{***}$
Chest circumference	$95.19\pm 4.06^{c}$	$97.24\pm 4.11^{\text{bc}}$	$98.34\pm 4.52^{\text{ab}}$	$101.50\pm 4.22^{a}$	0.001${}^{**}$
Front shin circumference	$12.00\pm 0.62^{b}$	$12.29\pm 0.67^{b}$	$12.22\pm 0.79^{b}$	$13.00\pm 1.05^{a}$	0.005${}^{**}$
Middle rump width	$23.67\pm 1.39^{b}$	$24.38\pm 1.03^{b}$	$25.22\pm 1.50^{a}$	$26.30\pm 1.70^{a}$	0.000${}^{***}$
Body length	$83.85\pm 2.63^{b}$	$84.38\pm 2.70^{b}$	$85.06\pm 2.73^{\text{ab}}$	$87.30\pm 2.26^{a}$	0.005${}^{**}$
Withers height	67.52±2.74	67.10±2.32	67.09±2.15	67.30±2.54	0.889${}^{\text{NS}}$
Rump height	65.30±1.64	64.86±2.56	65.66±3.08	65.70±1.83	0.528${}^{\text{NS}}$
Gestational length	151.41±3.50	151.26±5.46	150.81±4.87	148.90±6.28	0.548${}^{\text{NS}}$
Lamb birth weight	4.14±0.88	4.10±0.67	4.03±0.54	4.44±0.52	0.504${}^{\text{NS}}$

${}^{*}$ Statistically significant at the P<0.05 level. ${}^{**}$ Statistically significant at the P<0.01 level. ${}^{***}$ Statistically significant at the P<0.001 level. ${}^{\text{NS}}$ Statistically not significant (P>0.05). Means that do not share a letter are significantly different (Tukey test, P<0.05).

As seen in Table 3, the regression coefficients of all variables were not significant. Furthermore, the regression equation was not found to be significant. The $R^{2}$ value of the is very low. In this study, BCS, age, live weight during the mating period, the various body measurements (chest circumference, middle rump width, body length, wither height, rump height and front shin circumference), lamb birth weight and sex did not show a significant impact on the gestational length of Karayaka sheep.

**Table 3:** Regression coefficients for gestational length. SE refers to standard error, and VIF refers to the variance inflation factor.

Term	Coeff.	SE coeff.	T value	*P* value	VIF
Constant	15.7	2.21	0.71	0.479	
Body condition score	-1.221	0.694	-1.76	0.082	1.75
Ewe age	-0.200	0.326	-0.61	0.540	1.51
Ewe body weight at mating	0.163	0.177	0.92	0.361	5.21
Chest circumference	0.194	0.160	1.21	0.230	2.87
Front shin circumference	-0.277	0.630	-0.44	0.661	1.22
Middle rump width	-0.011	0.426	-0.03	0.980	2.38
Body length	-0.317	0.192	-1.65	0.102	1.50
Withers height	-0.200	0.217	-0.92	0.359	1.50
Rump height	0.239	0.202	1.18	0.240	1.44
Lamb birth weight	-0.064	0.678	-0.09	0.925	1.20
$R^{2}=12.09\;\%$					

**Figure 1:**
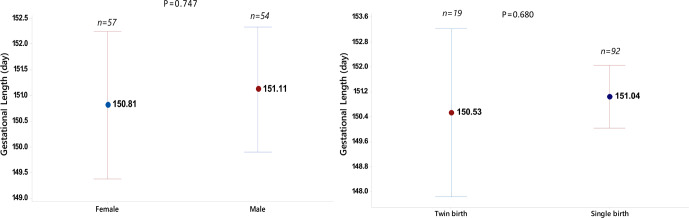
Interval plot showing the confidence interval of the gestational length for sheep with respect to the sex and birth type.

Gender and birth type also did not affect the gestational length in this sheep breed. However, it was observed that the gestational lengths of male offspring and animals from single offspring births were longer (Fig. 1). Gestational length was determined as 151.11 d for male offspring, 150.81 d for female offspring (P=0.747), 151.04 d for single offspring (non-multiple births) and 150.53 d for twins (P=0.680).

**Figure 2:**
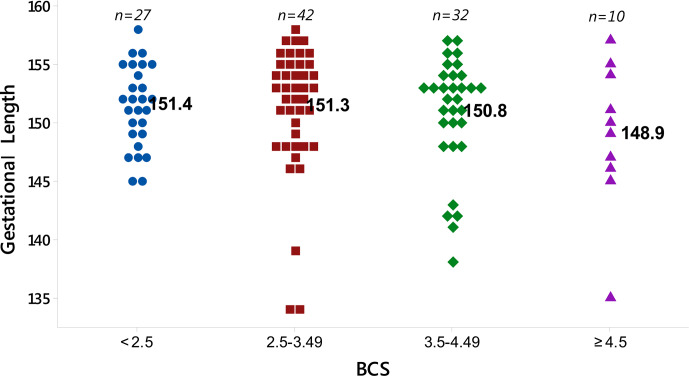
Individual value plot of gestational length for sheep in the four BCS groups.

The distribution of the gestational length with respect to the BCS is shown in Fig. 2. It was determined that BCS during the mating period did not affect the gestational length. However, as seen in Fig. 2, the lowest gestational length was seen in animals with a BCS ≥4.5. With respect to the four BCS groups, the gestational lengths were determined to be 151.4 d (BCS < 2.5), 151.3 d (BCS between 2.5 and 3.49), 150.8 d (BCS between 3.5 and 4.49) and 148.9 d (BCS≥4.5). This result shows that excessive fat during the mating period may affect the gestational length in Karayaka sheep.

## 4 Discussion

The finding that sex and birth type have no effect on the gestational length in Karayaka sheep is consistent with the study by Asmad et al. (2015). However, the findings of this study did not concur with Cam et al. (2018), who reported that the length of gestation was positively affected by the live weight and BCS of sheep. This divergence may be due to individual differences stemming from the farm, region and animals used in the studies.

In the province of Tokat, the rate of infertility, the birth rate, the twinning rate, the abortion rate and the number of lambs of per ram and per ewe in Karayaka sheep were 22.7 %, 73.0 %, 3.3 %, 2.1 %, 0.75 and 1.03 respectively (Belgüzar, 2011). These values diverge from the reports by Belgüzar (2011) and Cam et al. (2017), which may be due to regional differences and breeding practices.

The findings in this study that the gestational length in sheep was not affected by age, BCS, body measurements, sex, birth weight or birth type (P>0.05) were also different from what has been reported in many other studies in the literature (Ates et al., 2003; Ahmad and Khan, 2008; Cedden, 2002; Koyuncu et al., 2001; Koyuncu and Duru, 2003; Odabaşıoğlu et al., 1996). The average gestational length of Karacabey Merino sheep was found to be 150.97±0.054 d. The duration of gestation was significantly affected (P<0.01) by the year of gestation, the sex of the lamb and the mother's age, and the birth type was significant (P<0.05). The gestational period of 6-year-old mothers was found to be longer than the gestational length of 2-, 3-, 4- and 5-year-old mothers. The phenotypic correlation coefficient between birth weight and gestational length was reported to be 0.077±0.028 (P<0.05) (Koyuncu and Duru, 2003). The results of this study differ from those of Koyuncu and Duru (2003), and this difference may stem from genotype, business, and regional differences.

The mean gestational length was 150.273±0.240 d in Morkaraman sheep and 148.604±0.300 d in crosses. The effect of birth type, maternal age and sex on the duration of pregnancy was found to be insignificant (P>0.05) (Ateş et al., 2003). The mean gestational period of Konya Merino sheep was found to be 152.7±0.25 d, and year, age of the female, birth type and birth weight of the lamb affected the gestational length. It was determined that the gestational length (153.7±0.73) was longer for triple births than for twins, and the gestational length for twin births (152.8±0.16) was longer than for single births (151.6±0.22) (Öztürk and Aktaş, 1996). These results are similar to those reported by Ates et al. (2003), Öztürk and Aktaş (1996), Iyiola-Tunji et al. (2010), and Öztürk and Aktaş (1996).

The effect of age on the BCS was significant (P<0.05) in Karayaka sheep in this study: the BCS decreased with increasing age. This finding is not compatible with the publication by Türkyılmaz et al. (2017). It has been reported that Karayaka sheep with a BCS between 2.5 and 4.0 during the mating period have high reproductive performance (Cam et al., 2018), and our findings concur. The result obtained here regarding the BCS of sheep during the mating period was higher than the value reported by Özdemir (2008). In our study, it was found that the BCS was related to the body weight and chest circumference of animals, which was consistent with the findings of Worku (2019). The chest circumference and the BCS can provide information about the bodily reserves available to the sheep. This information is then easily utilized by the breeder. In Karayaka sheep, the findings regarding the BCS obtained before the mating period are similar to those reported by Thompson and Meyer (1994).

The finding in this study that the effects of the type of birth, the mother's age and the sex on the length of pregnancy were insignificant is consistent with the results of Ates et al. (2003).

Differences between the literature and the results of this study may be due to genotype, management, animal nutrition, breeders' practices and regional differences.

## 5 Conclusions

It was determined that the length of pregnancy did not change according to physical characteristics. However, it was observed that the gestational lengths of male and single offspring births were longer. The lowest gestational length was seen in animals with a BCS ≥4.5. It was determined that the BCS of the Karayaka sheep during the mating period was between 2.5 and 4.5. This result shows that the bodily reserves are good. Thus, it is beneficial if the BCS of the sheep during the mating period is between 2.5 and 4. Excessive fattening of sheep may cause difficulty during birth. It is recommended that breeders apply body condition scoring regularly to ensure efficient use of feed sources in sheep breeding. Controlling the BCS, which is an indicator of the bodily reserves of the sheep during the mating period, can prevent reproductive efficiency problems that may arise from nutritional deficiencies.

## Data Availability

The data are available from the corresponding author upon request.
